# Relationship between immune nutrition index and all-cause and cause-specific mortality in U.S. adults with chronic kidney disease

**DOI:** 10.3389/fnut.2023.1264618

**Published:** 2023-12-14

**Authors:** Junlin Zhang, Xiang Xiao, Tianzhao Han, Yuping Liu, Ping Shuai

**Affiliations:** ^1^Department of Health Management and Institute of Health Management, Sichuan Provincial People’s Hospital, University of Electronic Science and Technology of China, Chengdu, China; ^2^Department of Nephrology, The Third People’s Hospital of Chengdu, Southwest Jiaotong University, Chengdu, China; ^3^Department of Nephrology, The First Affiliated Hospital of Chengdu Medical College, Chengdu, China

**Keywords:** immunonutrition, prognostic nutritional index, chronic kidney disease, all-cause mortality, cardiovascular disease

## Abstract

**Objective:**

The available evidence regarding the association of immune nutrition status with chronic kidney disease (CKD) is limited. Thus, the present study examined whether immunonutrition indices were associated with renal function and mortality among CKD individuals.

**Research design and methods:**

This study enrolled 6,099 U.S. adults with CKD from the NHANES 2005–2018 database. Participants were matched with National Death Index records until 31 December 2019 to determine mortality outcomes. The time-dependent receiver operating characteristic was utilized to identify the most effective index among the prognostic nutritional index (PNI), system inflammation score (SIS), Naples prognostic score (NPS), and controlling nutritional status (CONUT) for predicting mortality. Cox regression models were employed to evaluate the associations of immunonutrition indices with mortality in participants with CKD.

**Results:**

The PNI exhibited the strongest predictive power among the four indices evaluated and the restricted cubic spline analysis revealed a cutoff value of 51 for the PNI in predicting mortality. During a median follow-up of 72 months (39–115 months), a total of 1,762 (weighted 24.26%) CKD participants died from all causes. The Kaplan–Meier curve demonstrated a reduced risk of death for the subjects with a higher PNI compared to those in the lower group. Besides, after adjusting for multiple potential confounders, a higher PNI remained an independent predictor for lower risks of all-cause mortality (HR 0.80, 95%CI: 0.71–0.91, *p* < 0.001) and cardiovascular disease (CVD) mortality (HR 0.69, 95%CI: 0.55–0.88, *p* = 0.002) in individuals with CKD.

**Conclusion:**

In CKD, a higher PNI level was significantly associated with lower mortality from all causes and CVD. Thus, the clinical utility of this immunonutrition indicator may facilitate risk stratification and prevent premature death among patients with CKD.

## Highlights

- This is the first study to investigate the associations of several immunonutrition indices including the PNI, SIS, CONUT, and NPS, with mortality in U.S. adults with chronic kidney disease;
- The PNI was the best indicator for predicting mortality in CKD and the cutoff value was 51;
- PNI was positively associated with eGFR and lymphocyte-to-monocyte ratio (LMR), and negatively correlated with UACR and neutrophil-to-lymphocyte ratio (NLR) in CKD;
- PNI was an independent predictor for all-cause and CVD mortality in CKD individuals.

## 1. Introduction

Chronic kidney disease (CKD) is a prevalent and potentially devastating medical disorder that has increased to affect approximately 15% of the general population worldwide, posing a significant public health challenge and burden in the 21st century (1, 2). CKD has been recognized as a condition of gradual decline in renal function that can ultimately progress to end-stage renal disease (ESRD) and is confirmed to be closely related to morbidity and mortality (3). Unfortunately, even with the optimal current treatment strategies, a substantial number of individuals with CKD remain at high risk of mortality and cardiovascular morbidity. In view of this, more effective targets for monitoring and intervention of CKD have been urgently explored.

In clinical practice, hypertension, diabetes, hyperuricemia, and obesity have been proven to be related to the acceleration and mortality risk of CKD (4, 5). As new risk factors of CKD progression continue to emerge, the evaluation of this disorder should consider the immunonutrition conditions of the patients with CKD. Inflammatory processes and malnutrition have been evidenced to be implicated in the development of CKD (6, 7). Currently, four objective indices are commonly used to evaluate the immunonutritional state of an individual, including the prognostic nutritional index (PNI), system inflammation score (SIS), controlling nutritional status (CONUT), and Naples prognostic score (NPS) (8–10). These indices are evaluated by the following elements: lymphocyte count, neutrophil count, monocyte count, serum albumin, and total cholesterol, and have been linked to prognosis in patients with a variety of diseases (9, 11, 12). However, epidemiological evidence on the relationship of immunonutrition status with mortality from all-cause and cardiovascular disease in CKD patients is limited, and which index is more effective in distinguishing subjects with CKD at high risk of mortality is still uncertain.

Thus, the purpose of this study was to evaluate and validate in detail the associations of multiple biomarkers of immunonutrition with renal function and all-cause and cause-specific mortality among individuals with CKD based on this prospective cohort of the NHANES 2005-2018.

## 2. Materials and methods

### 2.1. Study population

This study was performed in the National Health and Nutrition Examination Survey (NHANES) circles: 2005–2006, 2007–2008, 2009–2010, 2011–2012, 2013–2014, 2015–2016, and 2017–2018, which is a study evaluating the nutritional status of U.S. residents on a national scale. The details of the data can be found on the website: https://www.cdc.gov/nchs/nhanes/index.htm. NHANES was conducted by the National Center for Health Statistics of the Centers for Disease Control and Prevention (CDC), and authorized by the institutional review board of the National Center for Health Statistics. Written informed consent was provided by each participant.

**Figure 1:**
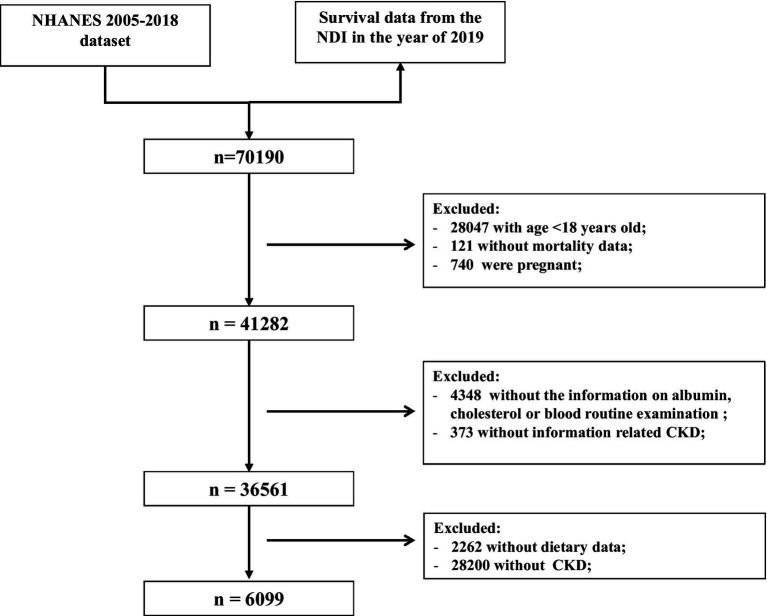
The flow chart of individual inclusion and exclusion in this study.

The exclusion criteria for this study were as follows (Figure 1): (1) aged <18 years old (*n* = 28,047); (2) those without mortality data (*n* = 121); (3) pregnant women (*n* = 740); and (4) missing information about serum albumin, cholesterol, blood routine examination (*n* = 4,348), UACR or eGFR (*n* = 373), and dietary data (*n* = 2,262). In total, 6,099 participants with CKD were included to evaluate the association of the immune-nutrition indices with all-cause and cardiovascular disease (CVD) mortality.

### 2.2. Clinical and laboratory data

Standardized questionnaires were utilized to collect data on age, sex, race, family income, education level, physical activity, smoking status, and disease status during household interviews. Based on their drinking status, participants were classified as either nondrinkers, low-to-moderate, or heavy drinkers (13). Physical activity was grouped into inactive, insufficiently active, or active groups (14). Body mass index (BMI) was calculated as weight (kg)/height (m)^2^. As criteria for diabetes, self-reporting diabetes history, hypoglycemic medications use, or satisfying the diagnostic criteria of the American Diabetes Association were considered (13, 15). CKD was defined as a urinary albumin-to-creatinine ratio (UACR) ≥ 30 mg/g and/or estimated glomerular filtration rate (eGFR) < 60 mL/min/1.73 m^2^ based on the recent guidelines (16).

Additionally, measurements of serum albumin, complete blood count, serum creatinine, UACR, triglycerides, total cholesterol, HDL cholesterol, LDL cholesterol, and serum uric acid were made at baseline. Stringent protocols were implemented during both the process of blood collection and subsequent analysis, with comprehensive documentation provided in the NHANES laboratory.

### 2.3. Assessment for immunonutrition indices

The PNI was determined by adding serum albumin (g/L) to 5 times the blood lymphocytes (17). Furthermore, the lymphocyte-to-monocyte ratio (LMR) and neutrophil-to-lymphocyte ratio (NLR) were calculated by finding the ratios of lymphocytes to monocytes and neutrophils to lymphocytes, respectively. The calculation methods for CONUT, SIS, and NPS are presented in Supplementary Table 1. The participants were then stratified into two groups based on their CONUT score: group 0 (score 0 and score 1) and group 1 (more than score 1). Participants were assigned to NPS-group 0 if they had a NPS score of 0, to NPS-group 1 for scores of 1 or 2, and to NPS-group 2 for scores of 3 or 4.

### 2.4. Study outcomes

In order to estimate mortality, the National Death Index for the year ending 31 December 2019 was linked with mortality data. The International Classification of Diseases-10 (ICD-10) was used to determine disease-specific death. CVD deaths included I00–I09, I11, I13, I20–I51, or I60–I69, and cancer deaths included C00–C97.

### 2.5. Statistical analyses

Given the complex multistage cluster survey design of the NHANES study, appropriate 14-year sampling weights were used based on the NHANES recommendation (18). The R version 4.1.1 (“Survey” package) was used in all the statistical analyses. Continuous variables were shown as means (standard errors) and categorical variables as weighted percentages. The differences in the continuous datasets were compared using the weighted *t*-test. Categorical datasets were analyzed by a weighted chi-squared test. To evaluate a dose–response association between PNI levels and mortality, restricted cubic spline regression with five knots was conducted. The Kaplan–Meier curves were used to examine the associations of PNI levels with all-cause, CVD, and cancer mortality in the CKD population. The Cox proportional hazards model was conducted to evaluate the hazard ratios (HRs) and 95% CIs for the relationship between PNI levels and all-cause and cause-specific death. In the multivariate models, we adjusted for age, sex, race, education level, family income to poverty ratio, BMI, smoking status, alcohol status, and physical activity in model 2. In model 3, we further adjusted for UACR, eGFR, diabetes, hypertension, CVD, and hyperuricemia. Stratified analyses were also performed by age, sex, race, BMI, smoking status, diabetes, hypertension, hyperuricemia, albumin, and eGFR. Statistical significance was defined as *p* < 0.05.

## 3. Results

### 3.1. Features of the study population with or without CKD

A total of 34,299 NHANES participants with or without CKD were included and their baseline characteristics were presented in Supplementary Table 2. The average age was 46.72 (0.26) years old, with males accounting for 48.95% of the population. The CKD prevalence was 14.33%, with those with CKD tending to be older and to have lower PNI [52.32 (0.23) vs. 53.92 (0.06)] and LMR values and higher NLR, CONUT, SIS, and NPS values relative to those without CKD. No significant difference was found in lymphocyte count. We also identified higher BMI and UACR, and lower eGFR and serum albumin in the participants with CKD. The overall prevalence of hyperuricemia, DM, hypertension, CVD, and cancer was 17.81, 13.71, 37.32, 8.58, and 9.64%, respectively, with the participants with CKD appearing to be at higher risk. People with CKD tended to have lower education levels, physical activity, and family income-to-poverty ratio and were more likely to be former smokers and non-drinkers.

### 3.2. Relationship between immunonutrition indices and CKD

**Figure 2:**
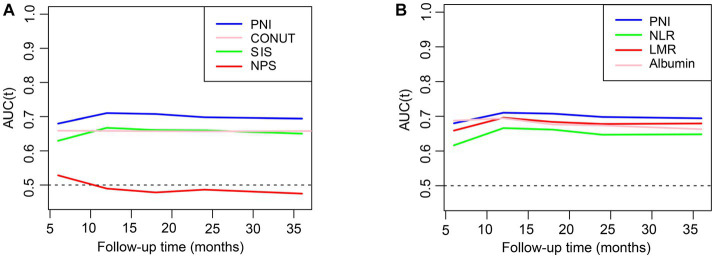
The relationships of inflammation and nutrition status with mortality in CKD. **(A,B)** The prediction of different indices for all-cause mortality evaluated with time-dependent receiver operating characteristic (td-ROC) curve. PNI, prognostic nutritional index; CONUT, controlling nutritional status: SIS, system inflammation score; NPS, Naples prognostic score; NLR, neutrophil-to-lymphocyte ratio; LMR, lymphocyte-to-monocyte ratio.

**Figure 3:**
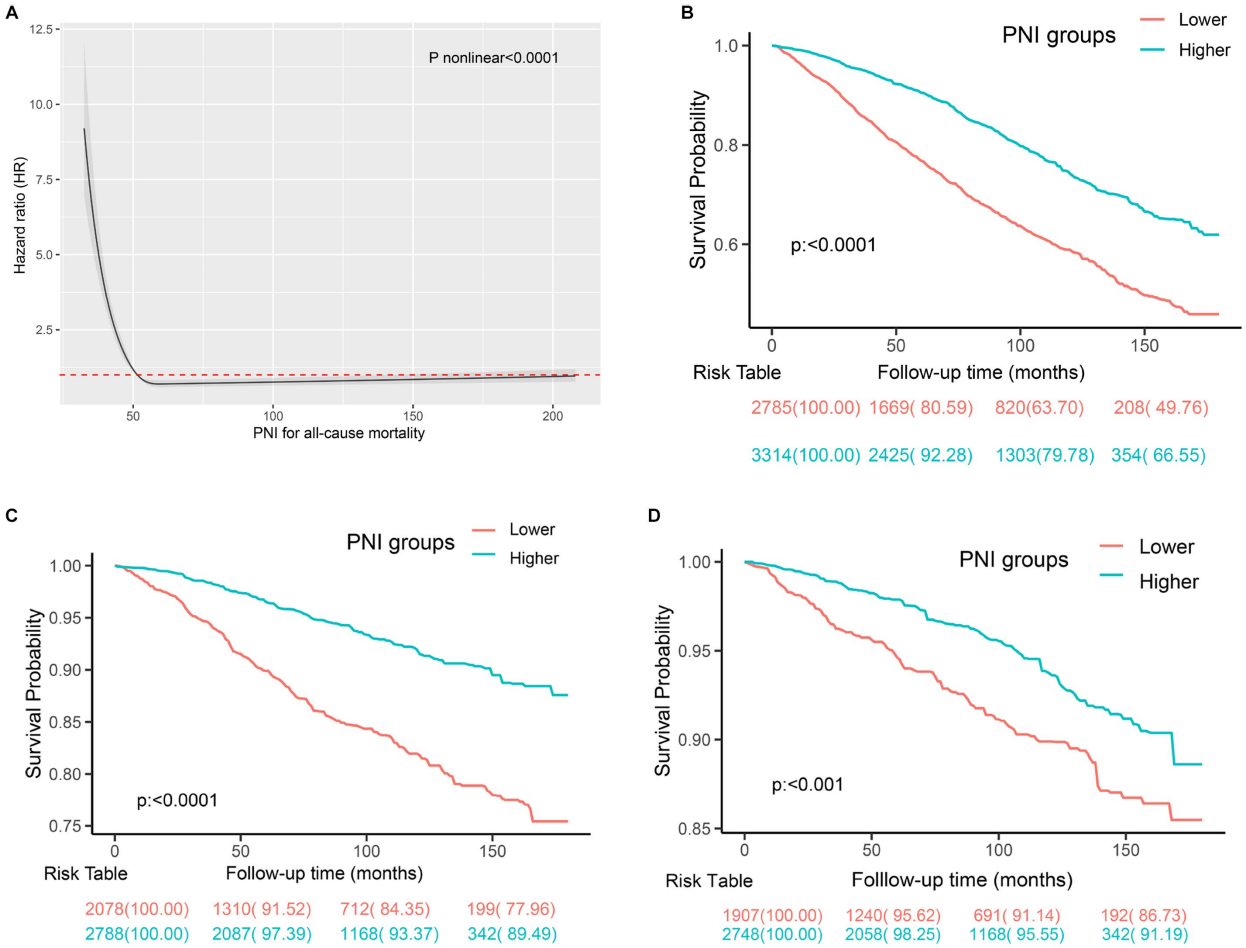
The prediction of mortality using the PNI in individuals with CKD. **(A)** The spline curve of PNI to predict mortality in CKD individuals using cox regression analysis. **(B)** Kaplan–Meier curves of all-cause mortality in participants with CKD with different PNI levels. **(C)** Kaplan–Meier curves of CVD mortality in the participants with CKD with different PNI levels. **(D)** Kaplan–Meier curves of cancer mortality in the participants with CKD with different PNI levels.

The estimated relationships between immunonutrition indices and all-cause mortality outcomes in people with CKD using time-dependent receiver operating characteristics are shown in Figure 2. The findings revealed that the PNI exhibited the highest predictive power for mortality among the SIS, CONUT, NPS, albumin, NLR, and LMR. We then employed the restricted cubic spline to explore the association between the PNI and mortality, and no linear association was observed (Figure 3A, p nonlinear <0.0001). Furthermore, the cutoff value for the PNI (the mortality risk to reach a nadir) in predicting the mortality in CKD individuals was determined to be 51, leading to the division of the cohort into two groups: higher group (PNI ≥ 51) and lower group (PNI < 51).

Table 1 summarizes the baseline features for the 6,099 participants with CKD. Significant differences between the higher and lower groups were observed for age, race, physical activity, smoking status, and alcohol use, as well as for the prevalence of hyperuricemia, hypertension, diabetes, CVD, and cancer. Mean baseline eGFR, protein intake, total cholesterol, serum albumin, and LMR were higher, and UACR and NLR were lower in participants with a higher PNI compared to those with a lower PNI. The general linear model with adjustments for age, sex, and race indicated that higher PNI level was negatively associated with UACR, blood urea nitrogen, and NLR, and positively related with eGFR and LMR at baseline (*p* < 0.05, Supplementary Table 3).

**Table 1:** Clinical features of the participants with CKD in different PNI groups.

Variable	Lower PNI (*n* = 2,785)	Higher PNI (*n* = 3,314)	*p*-value
Age (years)	65.18 (0.46)	56.86 (0.48)	<0.0001
Sex (male, %)	43.61	42.10	0.43
Race (%)			0.01
Non-Hispanic White	71.93	68.07	
Others	28.07	31.93	
BMI (kg/m^2^)	30.53 (0.23)	29.96 (0.17)	0.03
BMI category (%)			0.66
Normal (<25)	24.19	25.48	
Overweight (25–30)	30.39	29.38	
Obesity (≥30)	45.41	45.14	
Education (%)			0.29
Less than high school	8.93	8.99	
High school or equivalent	40.49	38.08	
College or above	50.57	52.94	
Family income-poverty ratio (%)			0.45
≤1.0	15.87	16.55	
1–3	45.27	43.02	
>3.0	38.85	40.43	
Physical activity (%)			<0.0001
Inactive	41.16	31.70	
Insufficiency	18.58	18.67	
Active	40.26	49.63	
Smoking (%)			<0.0001
Non-smoker	51.33	51.51	
Former smoker	36.81	29.16	
Current smoker	11.86	19.32	
Alcohol use (%)			<0.0001
Nondrinker	40.45	34.65	
Low-to moderate drinker	50.00	49.90	
Heavy drinker	9.55	15.46	
Energy intake (kcal/d)	1868.56 (19.94)	1932.33 (24.53)	0.05
Protein intake (g/d)	70.82 (0.93)	75.90 (1.12)	<0.001
Hyperuricemia (%)	35.91	32.30	0.03
Hypertension (%)	72.99	63.35	<0.0001
Diabetes (%)	37.74	32.40	0.01
Triglyceride (mg/dL)	128.32 (3.29)	159.06 (4.59)	<0.0001
HDL-C (mg/dL)	53.48 (0.39)	52.20 (0.51)	0.04
LDL-C (mg/dL)	104.25 (1.27)	113.23 (1.43)	<0.0001
UACR (mg/g)	273.61 (22.35)	119.11 (6.14)	<0.0001
eGFR (ml/min/1.73m^2^)	65.30 (0.72)	78.76 (0.72)	<0.0001
eGFR categories			<0.0001
<60	57.71	40.46	
60–90	20.33	21.05	
≥90	21.96	38.50	
CVD (%)	33.29	17.88	<0.0001
Cancer (%)	22.89	15.22	<0.0001
Serum albumin (g/L)	39.27 (0.09)	43.31 (0.08)	<0.0001
Total cholesterol (mg/dL)	186.46 (1.30)	197.81 (0.99)	<0.0001
Lymphocytes (×10^9^/L)	1.57 (0.01)	2.58 (0.07)	<0.0001
NLR	3.11 (0.04)	2.10 (0.03)	<0.0001
LMR	3.07 (0.04)	4.25 (0.05)	<0.0001
PNI	47.13 (0.09)	56.21 (0.34)	<0.0001
CONUT group			<0.0001
≤1	55.62	89.34	
>1	44.38	10.66	
SIS			<0.0001
0	4.82	29.62	
1	55.44	66.12	
2	39.75	4.26	
NPS group			<0.0001
0	3.58	0.93	
1	52.55	63.34	
2	43.88	35.73	
Decreased (%)	31.73	18.66	<0.0001

BMI, body mass index; UACR, urinary albumin creatinine ratio; e-GFR, estimated glomerular filtration rate; CVD, cardiovascular disease; NLR, neutrophil to lymphocyte ratio; LMR, lymphocyte to monocyte ratio; PNI, prognostic nutritional index; CONUT, controlling nutritional status; SIS, system inflammation score; NPS, Naples prognostic score. Data is presented as the mean ± standard error (SE) or weighted percentage %.

### 3.3. Association between the PNI and mortality in CKD

**Figure 4:**
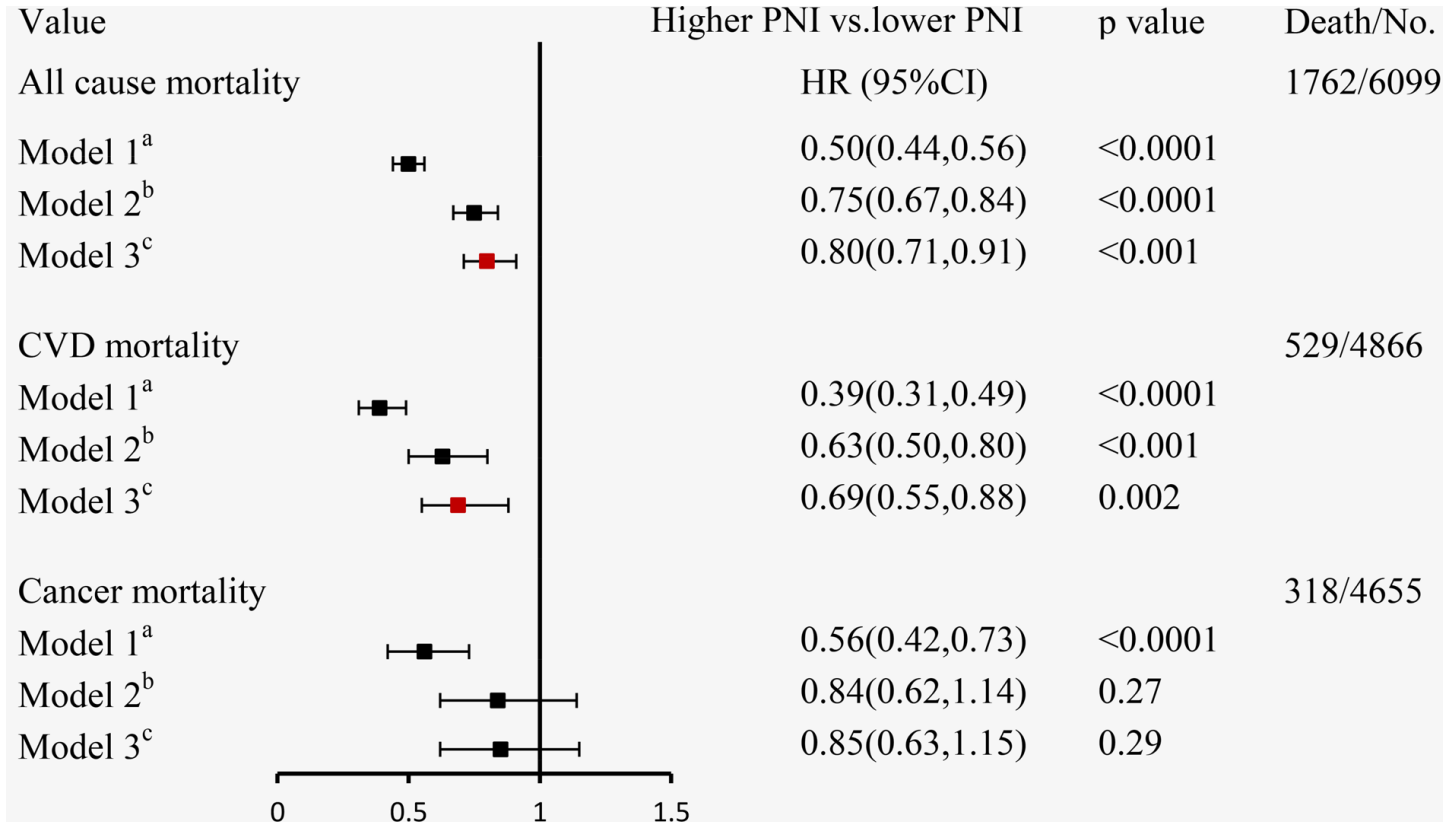
The relationship between PNI and mortality using Cox regression analysis in individuals with CKD. ^a^Model 1: unadjusted; ^b^Model 2: adjusted for age, sex, race/ethnicity, BMI, education level, family income-poverty ratio, smoking status, alcohol status, and physical activity; ^c^Model 3: further adjusted (from Model 2) for UACR, eGFR, diabetes, hypertension, CVD, and hyperuricemia.

**Figure 5:**
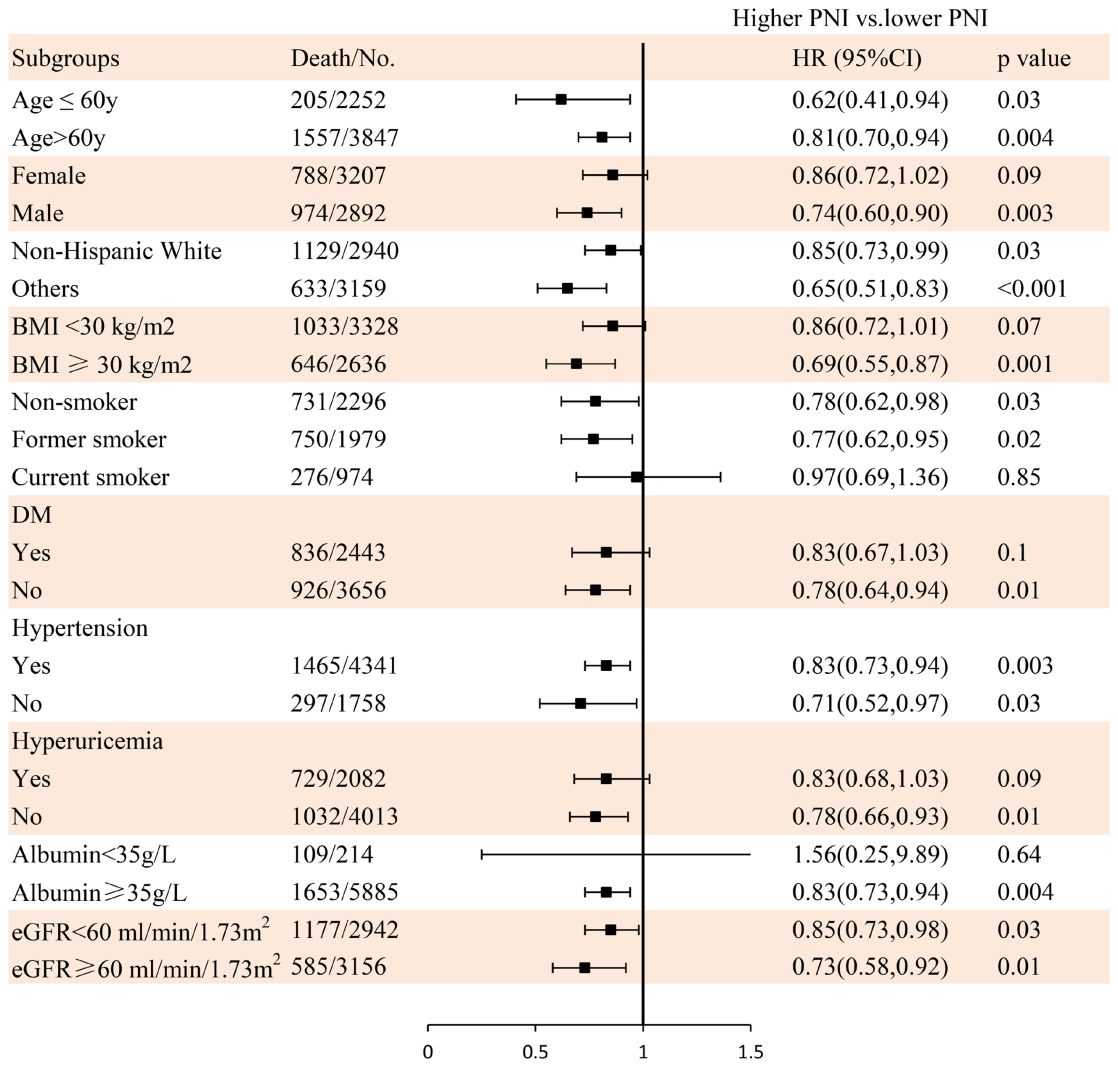
The relationship between PNI and all-cause mortality using Cox regression analysis in individuals with CKD in subgroups. Adjusted for age, sex, race/ethnicity, BMI, education level, family income-poverty ratio, smoking status, alcohol status, physical activity, protein intake, UACR, eGFR, diabetes, hypertension, CVD, and hyperuricemia.

During a mean follow-up time of 72 months (39–115 months) in 6,099 participants with CKD at baseline, we identified 1,762 deaths from any cause, 529 deaths from CVD, and 318 deaths from cancer. The Kaplan–Meier curves demonstrated that subjects with a higher PNI had significantly lower incidences of all-cause mortality (Figure 3B), CVD mortality (Figure 3C), and cancer mortality (Figure 3D) compared to those in the lower PNI group (log-rank test, *p* < 0.001). Cox regression analyses of PNI with all-cause, CVD, and cancer mortality in participants with CKD are summarized in Figure 4. In the univariate analysis (model 1), subjects with a higher PNI had decreased risks of all-cause (HR 0.50, 95%CI: 0.44–0.56, *p* < 0.0001), CVD (HR 0.39, 95%CI: 0.31–0.49, *p* < 0.0001), and cancer death (HR 0.56, 95%CI: 0.42–0.73, *p* < 0.0001). The multivariate-adjusted HRs and 95% CIs (model 3) for the higher PNI group were 0.80 (0.71–0.91) for all-cause mortality (Supplementary Table 4), 0.69 (0.55–0.88) for CVD mortality, and 0.85 (0.63–1.15) for cancer mortality. In the stratified analysis, these results were similar by age, race, hypertension, and eGFR (Figure 5). However, PNI level was not related to all-cause mortality in females, those with a BMI <30 kg/m^2^, DM, hyperuricemia or albumin <35 g/L, and current smokers with CKD after adjustment for multiple confounders.

### 3.4. Other immunonutrition indices and mortality in CKD

We then proceeded to perform a Cox analysis to examine the association between mortality and various inflammatory and nutritional indices, including albumin, total cholesterol, lymphocytes, NLR, LMR, CONUT, SIS, and NPS (Table 2). In the multivariate Cox proportional hazards analysis, serum albumin, NLR, LMR, CONUT, and SIS were independently related to all-cause and CVD mortality. Additionally, the serum albumin, lymphocytes, NLR, and SIS were identified as independent predictors for cancer-related death. Lymphocytes were also found to be associated with CVD death, but not with all-cause mortality.

**Table 2:** The relationships between immunonutrition status and mortality in CKD.

Variable	All-cause		CVD mortality		Cancer mortality	
	HR (95%CI)	*p* value^*^	HR (95%CI)	*p* value^*^	HR (95%CI)	*p* value^*^
Serum albumin (g/L)	0.94 (0.92, 0.96)	<0.0001	0.91 (0.88, 0.95)	<0.0001	0.95 (0.90, 0.99)	0.03
Total cholesterol (mg/dL)	1.00 (1.00, 1.00)	0.93	1.00 (1.00, 1.00)	0.50	1.00 (1.00, 1.00)	0.51
Lymphocytes (×10^9^/L)	1.01 (1.00, 1.02)	0.18	0.79 (0.63, 0.99)	0.04	1.01 (1.00, 1.02)	<0.001
NLR	1.11 (1.06, 1.16)	<0.0001	1.12 (1.06, 1.19)	<0.001	1.14 (1.06, 1.22)	<0.001
LMR	0.94 (0.89, 0.996)	0.03	0.82 (0.72, 0.93)	0.001	1.03 (0.99, 1.06)	0.15
CONUT	1.15 (1.07, 1.22)	<0.0001	1.21 (1.09, 1.34)	<0.001	1.15 (0.99, 1.33)	0.06
SIS	1.42 (1.24, 1.63)	<0.0001	1.74 (1.40, 2.15)	<0.0001	1.57 (1.14, 2.17)	0.01
NPS	0.95 (0.87, 1.03)	0.18	0.92 (0.79, 1.06)	0.26	0.98 (0.80, 1.21)	0.86

^*^Adjusted for adjusted for age, sex, race/ethnicity, BMI, education level, family income-poverty ratio, smoking status, alcohol status, physical activity, protein intake, UACR, eGFR, diabetes, hypertension, CVD, and hyperuricemia.

## 4. Discussion

CKD remains a worldwide challenge with disappointing long-term outcomes, and the identification of prognostic factors for mortality has become an essential topic of recent research. To our knowledge, this is the first prospective study to investigate the independent associations of various inflammatory and nutritional indices with all-cause and cause-specific mortality in a nationally representative cohort of individuals with CKD. Our findings indicate that the prevalence of CKD is linked to lower PNI and higher SIS, NPS, and CONUT levels. Additionally, PNI is the most effective index for predicting all-cause mortality in individuals with CKD and is associated with UACR, eGFR, NLR, and LMR. Furthermore, a higher PNI level is significantly correlated with a lower risk of all-cause and CVD mortality, but not with cancer-related mortality. These findings support the clinical utility of PNI which may facilitate the risk stratification and timely intervention for premature death among individuals with CKD.

The PNI is a reliable indicator of the immunonutritional status of individuals, which is based on lymphocyte and albumin levels. Recent findings have not only substantiated the potential of the PNI as a prognostic indicator for various malignancies (19–22), COVID-19 (9, 23), coronary artery disease (24), and acute renal dysfunction (25), but also shown that a lower PNI has been linked to increased mortality risk in elderly individuals with CKD (26), and diabetic kidney disease (DKD) (27). In this study, we further corroborated the significant correlation between the PNI and CKD in the general population. Specifically, the optimal PNI cutoff point was 51, which had a superior predictive effect compared to other immunonutrition indices in CKD.

CONUT, which incorporates multiple parameters, has been shown to predict not only malnutrition but also the severity of inflammation, significantly correlating with mortality among patients with peritoneal dialysis (28). Furthermore, Takagi et al. (29) found that CONUT was closely related to protein-energy wasting (PEW) and could predict infectious disease mortality in 311 patients with CKD at dialysis. Furthermore, Tsuda et al. (30) reported that both the PNI and CONUT were significantly related to prior CVD in 2,751 CKD subjects. However, the studies on the association of SIS with CKD were limited. In this study, we found that CONUT and SIS were not only related to the prevalence of CK but also associated with mortality from all-cause and CVD in the participants with CKD. These findings suggested that CONUT and SIS might aid in the prediction of individual patient risk, identification of malnourished or inflamed patients, and initiation of prompt nutritional and anti-inflammatory interventions, which could contribute to reducing the high mortality rates in CKD populations.

In recent years, there has been a notable surge in research exploring the predictive value of inflammatory and nutritional markers, and their correlation with mortality in patients with CKD. Serum albumin, as one factor of the PNI, is an important indicator reflecting individual nutrition and has antioxidant, anti-inflammatory, and antiplatelet aggregation effects (31). Serum albumin level has been reported to be independently related to renal prognosis in patients with diabetic kidney disease (32, 33). Other studies found that serum albumin was closely associated with CVD including heart failure, stroke, and coronary artery disease, and overall survival in CKD patients (34, 35). Blood lymphocytes, as the other factor of the PNI, have been reported to be significantly related to all-cause mortality in diabetes and cardiovascular diseases (36, 37). Furthermore, among the analyzed markers, the NLR and MLR had shown a better predictive ability for survival in patients with cancer (38) and acute kidney injury (AKI) in patients with severe acute pancreatitis (39) compared to the classic inflammatory factors such as high-sensitivity CRP and TNF-α. Several studies suggested that increased NLR and MLR affected glomerular injury and renal function in individuals with DKD (40–44). Zhang et al. (45) and Liao et al. (46) discovered a close relationship between higher NLR and MLR levels and all-cause mortality in hemodialysis patients. In this study, the findings also demonstrated that serum albumin, the NLR, and the MLR were independently related to all-cause and CVD mortality in patients with CKD. However, the PNI, which incorporates albumin and lymphocytes, was the most reliable indicator for predicting all-cause mortality in individuals diagnosed with CKD.

Malnutrition is not uncommon in individuals with CKD, and often along with obesity, which causes dual harm to patients (30). Tsuda et al. (30) found that a lower PNI was independently related to CVD in patients with a higher BMI, whereas this relationship did not exist in individuals with a low BMI. Chien et al. (47) also suggested that compared with lean individuals with malnutrition, obese participants with undernutrition had a higher prevalence of CVD in Taiwanese adults. In this study, we also found that a higher PNI was significantly associated with a decreased mortality risk in individuals with a higher BMI (≥30 kg/m^2^), but not in participants with a lower BMI, which was similar to the findings of Tsuda et al. and Chien et al.

Intervention research on malnutrition in patients with CKD is currently limited. Dietary interventions are the cornerstone in the prevention and management of malnutrition in individuals with CKD and are closely associated with PEW. In our study, we observed that participants with CKD had lower energy and protein intake, as well as a lower PNI compared to those without CKD. However, our Cox regression analysis revealed that diet protein intake was not associated with the risk of mortality in individuals with CKD. Whether individuals with CKD (eGFR 30–60 mL/min/1.73 m^2^) should adhere to a low protein diet (LPD) (48, 49) of 0.55–0.6 g/kg/day (without diabetes) or 0.6–0.8 g/kg/day (with diabetes) to improve outcomes and quality of life remains controversial. However, restricted protein intake was considered to play an important role in malnutrition, PEW, sarcopenia, and comorbidities in older individuals with CKD (50, 51). Thus, a recent review endorsed by the European Society for Clinical Nutrition and Metabolism (ESPEN) and the European Renal Nutrition group of the European Renal Association (ERN-ERA) suggests guiding clinical decisions for elderly patients with CKD with an individual risk–benefit assessment (52, 53).

Nutrition, immunity, and chronic inflammation have been proven to be closely related. Low-grade systemic inflammation is a well-established cause of poor outcomes in CKD and CVD (54, 55)., which could potentially lead to protein malnutrition and immune dysfunction, further exacerbating inflammation and perpetuating a detrimental cycle that accelerates the progression of disease (56–58). These findings may partly explain why the PNI is the best indicator of mortality in CKD. Interventions targeting both nutrition and immuno-inflammation may reduce the risk of cardiovascular and all-cause mortality in CKD. Reducing inflammation through dietary interventions appears to be a promising possibility. However, the most suitable diet model to effectively reduce inflammation and improve protein nutrition in humans remains uncertain.

This study’s advantages include its prospective study design and the utilization of a nationally representative U.S. adult CKD sample, thereby enhancing the generalizability of our findings. Furthermore, the comprehensive data obtained from the NHANES allowed us to adjust for numerous confounders, such as dietary and lifestyle factors and socioeconomic status. However, several limitations should also be considered. First, it should be noted that the diagnosis of CKD in this study was established through a single assessment of eGFR and UACR, rather than a longitudinal observation period of 3 months. Nevertheless, the consistency of the results with the prevalence of CKD suggests a degree of reliability. Second, certain potential confounding factors, such as medication interventions, were not accounted for in the analysis, which may have influenced the study outcomes. Third, our primary focus was on the indicators that can simultaneously reflect both nutritional and immune functions. Consequently, we did not further investigate individual inflammatory factors, such as cytokines, that exclusively reflect immune function. The selected indicators for this study have advantages in terms of accessibility and holistic assessment. However, they also have disadvantages such as limited specificity and lack of mechanistic insights.

In brief, our study has evinced a noteworthy correlation between elevated PNI levels and reduced all-cause and cardiovascular disease mortality in a representative cohort of individuals with CKD in the United States. These results lend credence to the potential clinical applicability of the PNI as a tool for risk stratification and timely intervention to avert premature mortality in this population.

## Data availability statement

The datasets presented in this study can be found in online repositories. The names of the repository/repositories and accession number(s) can be found below: https://www.cdc.gov/nchs/nhanes/index.htm.

## Ethics statement

The studies involving humans were approved by NCHS Research Ethics Review Board. The studies were conducted in accordance with the local legislation and institutional requirements. The participants provided their written informed consent to participate in this study.

## Author contributions

JZ: Conceptualization, Funding acquisition, Methodology, Writing – original draft, Project administration. XX: Data curation, Methodology, Supervision, Visualization, Writing – original draft. TH: Investigation, Software, Validation, Formal analysis, Resources, Writing – review & editing. YL: Supervision, Validation, Writing – review & editing. PS: Conceptualization, Funding acquisition, Validation, Writing – review & editing.
