# Supplementary material for: Relationship between immune nutrition index and all-cause and cause-specific mortality in U.S. adults with chronic kidney disease
Source: Front Nutr. 2023 Dec 14;10:1264618. doi: 10.3389/fnut.2023.1264618 (PMC10752924; doi:10.3389/fnut.2023.1264618)
Supplement: Supplementary file 1 [file Table_1.docx]

**Supplementary Table 1. The methods for the CONUT, SIS, and NPS calculations**

| Indices | Elements |  | Scores |
| --- | --- | --- | --- |
| CONUT 0-12 | Serum albumin (g/L) | ≥35 | 0 |
|  |  | 30-34 | 2 |
|  |  | 25-29 | 4 |
|  |  | <25 | 6 |
|  | Total cholesterol (mg/dl) | >180 | 0 |
|  |  | 140-180 | 1 |
|  |  | 100-139 | 2 |
|  |  | <100 | 3 |
|  | Total lymphocytes (×10^9^/L) | >1.6 | 0 |
|  |  | 1.2-1.6 | 1 |
|  |  | 0.8-1.199 | 2 |
|  |  | <0.8 | 3 |
| SIS 0-2 | serum albumin level > 40 g/L  and LMR level ≥ 4.44 |  | 0 |
|  | serum albumin level≤4 0 g/L  or LMR level < 4.44 |  | 1 |
|  | serum albumin level≤40 g/L  and LMR level < 4.44 |  | 2 |
| NPS 0-4 | Serum albumin | >40g/L | 0 |
|  |  | ≤40 g/L | 1 |
|  | LMR | ≥4.44 | 0 |
|  |  | <4.44 | 1 |
|  | NLR | ≤2.96 | 0 |
|  |  | >2.96 | 1 |
|  | Total cholesterol (mg/dl) | >180 | 0 |
|  |  | ≤180 | 1 |

Note: CONUT: controlling nutritional status; SIS: system inflammation score; NPS: Naples prognostic score.

NLR: neutrophil to lymphocyte ratio; LMR: lymphocyte to monocyte ratio.

**Supplementary Table 2. Clinical features of the participants with or without CKD**

| **Variable** | **All**  **(n=34299)** | **Non-CKD**  **(n=28200)** | **CKD**  **(n=6099)** | **p-value** |
| --- | --- | --- | --- | --- |
| **Age (years)** | 46.72(0.26) | 44.43(0.24) | 60.42(0.33) | < 0.0001 |
| **Gender (male, %)** | 48.95 | 49.99 | 42.75 | < 0.0001 |
| **Race (%)** |  |  |  | 0.001 |
| **Non-Hispanic White** | 67.33 | 66.93 | 69.72 |  |
| **Others** | 32.67 | 33.07 | 30.28 |  |
| **BMI (kg/m^2^)** | 28.91(0.09) | 28.69(0.08) | 30.20(0.15) | < 0.0001 |
| **BMI category (%)** |  |  |  | < 0.0001 |
| **Normal (<25)** | 30.44 | 31.60 | 24.94 |  |
| **Overweight (25-30)** | 32.43 | 33.12 | 29.81 |  |
| **Obesity (≥30)** | 36.44 | 35.28 | 45.25 |  |
| **Education (%)** |  |  |  | < 0.0001 |
| **Less than high school** | 5.03 | 4.57 | 8.96 |  |
| **High school or equivalent** | 32.64 | 32.91 | 39.12 |  |
| **College or above** | 58.85 | 62.52 | 51.92 |  |
| **Family income-poverty ratio (%)** |  |  |  | < 0.0001 |
| **≤1.0** | 14.02 | 14.81 | 16.26 |  |
| **1-3** | 33.16 | 34.09 | 43.99 |  |
| **>3.0** | 46.21 | 51.10 | 39.76 |  |
| **Physical activity (%)** |  |  |  | < 0.0001 |
| **Inactive** | 21.50 | 19.12 | 35.75 |  |
| **Insufficiency** | 17.38 | 17.16 | 18.63 |  |
| **Active** | 61.12 | 63.71 | 45.62 |  |
| **Smoking (%)** |  |  |  | < 0.0001 |
| **Non-smoker** | 53.95 | 55.68 | 51.44 |  |
| **Former smoker** | 24.14 | 23.32 | 32.46 |  |
| **Current smoker** | 19.89 | 21.01 | 16.11 |  |
| **Alcohol use (%)** |  |  |  | < 0.0001 |
| **Nondrinker** | 22.32 | 22.22 | 37.05 |  |
| **Low-to moderate drinker** | 49.22 | 54.24 | 49.94 |  |
| **Heavy drinker** | 20.24 | 23.54 | 13.01 |  |
| **Energy intake (kcal/d)** | 2158.70(8.32) | 2201.15(8.77) | 1905.03(16.23) | < 0.0001 |
| **Protein intake (g/d)** | 83.40(0.39) | 85.02(0.42) | 73.73(0.75) | < 0.0001 |
| **Hyperuricemia (%)** | 17.81 | 15.13 | 33.84 | < 0.0001 |
| **Diabetes (%)** | 13.71 | 10.20 | 34.69 | < 0.0001 |
| **Hypertension (%)** | 37.32 | 32.27 | 67.48 | < 0.0001 |
| **Triglyceride (mg/dL)** | 125.44(1.24) | 122.32(1.39) | 144.22(2.91) | < 0.0001 |
| **HDL-C (mg/dL)** | 53.30(0.19) | 53.40(0.20) | 52.75(0.35) | 0.07 |
| **LDL-C (mg/dL)** | 113.81(0.47) | 114.62(0.52) | 108.85(0.97) | < 0.0001 |
| **UACR (mg/g)** | 32.61(1.54) | 7.72(0.06) | 184.29(10.49) | < 0.0001 |
| **eGFR (ml/min/1.73m^2^)** | 94.77(0.34) | 98.41(0.31) | 73.00(0.56) | < 0.0001 |
| **CVD (%)** | 8.58 | 6.22 | 24.54 | < 0.0001 |
| **Cancer (%)** | 9.64 | 8.54 | 18.53 | < 0.0001 |
| **Serum albumin (g/L)** | 42.82(0.05) | 43.03(0.05) | 41.58(0.08) | < 0.0001 |
| **Total cholesterol (mg/dL)** | 193.54(0.47) | 193.64(0.50) | 192.95(0.89) | 0.47 |
| **Lymphocytes (×10^9^/L)** | 2.17(0.01) | 2.18(0.01) | 2.15(0.04) | 0.48 |
| **NLR** | 2.17(0.01) | 2.11(0.01) | 2.53(0.03) | < 0.0001 |
| **LMR** | 4.09(0.02) | 4.15(0.02) | 3.74(0.04) | < 0.0001 |
| **PNI** | 53.69(0.07) | 53.92(0.06) | 52.32(0.23) | < 0.0001 |
| **CONUT group** |  |  |  | < 0.0001 |
| **≤1** | 82.98 | 84.34 | 74.90 |  |
| **>1** | 17.02 | 15.66 | 25.10 |  |
| **SIS** |  |  |  | < 0.0001 |
| **0** | 28.55 | 30.15 | 19.00 |  |
| **1** | 61.34 | 61.31 | 61.55 |  |
| **2** | 10.10 | 8.54 | 19.45 |  |
| **NPS group** |  |  |  | < 0.0001 |
| **0** | 1.09 | 0.92 | 2.06 |  |
| **1** | 60.67 | 60.99 | 58.72 |  |
| **2** | 38.25 | 38.09 | 39.22 |  |

Note: BMI: body mass index; HbA1c: glycosylated hemoglobin A1c; HOMA-RI: homeostasis model assessment of insulin resistance; DKD, diabetic kidney disease; e-GFR, estimated glomerular filtration rate; CVD: cardiovascular disease; NLR: neutrophil to lymphocyte ratio; LMR: lymphocyte to monocyte ratio; PNI: prognostic nutritional index; CONUT: controlling nutritional status: SIS: system inflammation score; NPS: Naples prognostic score. Data is presented as the mean ± standard error (SE) or weighted percentages.

**Supplementary Table 3. The associations between the PNI and the clinical characteristics of CKD**

| **Variables** | **Higher PNI vs. lower PNI** | |
| --- | --- | --- |
|  | **β*** | **P value** |
| **UACR** | -179.54(-231.99, -127.10) | <0.0001 |
| **eGFR** | 3.91(2.49, 5.33) | <0.0001 |
| **Blood urea nitrogen** | -0.61(-0.81, -0.41) | <0.0001 |
| **Serum uric acid** | -1.04(-7.95, 5.86) | 0.77 |
| **NLR** | -0.96(-1.06, -0.87) | <0.0001 |
| **LMR** | 1.05(0.91, 1.19) | <0.0001 |

Note: *Adjustment for age, sex, and race. UACR, urinary albumin-to-creatinine ratio. e-GFR, estimated glomerular filtration rate; NLR: Neutrophil lymphocyte ratio. LMR: lymphocyte to monocyte ratio; β, regression coefficient; CI, confidence interval.

**Supplementary Table 4. The relationship between the PNI and mortality using Cox regression analysis.**

| **Variable** | **Univariate analysis** | | **Model 3*** | | |
| --- | --- | --- | --- | --- | --- |
|  | **HR (95%CI)** | **P value** | | **HR (95%CI)** | **P value** |
| **Age (yrs)** | 1.07(1.06,1.08) | <0.0001 | | 1.07(1.06,1.08) | <0.0001 |
| **Gender (Male)** | 1.29(1.13,1.48) | <0.001 | | 1.40(1.20,1.64) | <0.0001 |
| **Race (vs. ohters)** |  |  | |  |  |
| Non-Hispanic White | 1.69(1.46,1.94) | <0.0001 | | 1.33(1.14,1.55) | <0.001 |
| **Education (vs. Less than high school)** |  |  | |  |  |
| High school or equivalent | 0.81(0.68,0.97) | 0.02 | | 1.09(0.89,1.34) | 0.39 |
| College or above | 0.57(0.47,0.69) | <0.0001 | | 1.19(0.97,1.48) | 0.10 |
| **Family income-poverty ratio (vs. <=1)** |  |  | |  |  |
| 1-3 | 1.25(1.07,1.46) | 0.01 | | 0.75(0.60,0.93) | 0.01 |
| >3 | 0.63(0.52,0.77) | <0.0001 | | 0.56(0.43,0.74) | <0.0001 |
| **Physical activity (vs. inactive)** |  |  | |  |  |
| Insufficiency | 0.43(0.36,0.51) | <0.0001 | | 0.60(0.49,0.74) | <0.0001 |
| Active | 0.32(0.27,0.38) | <0.0001 | | 0.53(0.44,0.63) | <0.0001 |
| **Smoking (vs. non-smoker)** |  |  | |  |  |
| Former smoker | 1.74(1.51,2.00) | <0.0001 | | 1.22(1.05,1.42) | 0.01 |
| Current smoker | 1.19(0.97,1.46) | 0.09 | | 1.71(1.36,2.16) | <0.0001 |
| **Alcohol use (vs. Nondrinker)** |  |  | |  |  |
| Low-to moderate drinker | 0.52(0.44,0.61) | <0.0001 | | 0.70(0.60,0.83) | <0.0001 |
| Heavy drinker | 0.44(0.35,0.57) | <0.0001 | | 1.16(0.83,1.62) | 0.39 |
| **BMI (vs. normal)** |  |  | |  |  |
| Overweight (25-30) | 0.97(0.82,1.15) | 0.69 | | 0.69(0.58,0.82) | <0.0001 |
| Obesity (≥30) | 0.88(0.74,1.05) | 0.17 | | 0.73(0.60,0.88) | 0.001 |
| **Protein intake (g/day)** | 0.99(0.99,1.00) | <0.001 | | 1.00(1.00,1.00) | 0.92 |
| **UACR (mg/g)** | 1.00(1.00,1.00) | <0.0001 | | 1.00(1.00,1.00) | <0.0001 |
| **eGFR (ml/min/1.73m^2^)** | 0.97(0.97,0.98) | <0.0001 | | 1.00(0.99,1.00) | 0.46 |
| **Hypertension (yes vs. no)** | 2.94(2.52,3.44) | <0.0001 | | 1.32(1.13,1.54) | <0.001 |
| **DM (yes vs. no)** | 1.79(1.59,2.02) | <0.0001 | | 1.27(1.10,1.46) | <0.001 |
| **Hyperuricemia (yes vs. no)** | 1.60(1.41,1.81) | <0.0001 | | 1.14(0.97,1.34) | 0.10 |
| **CVD (yes vs. no)** | 3.12(2.72,3.58) | <0.0001 | | 1.34(1.15,1.56) | <0.001 |
| **Higher PNI (vs lower PNI)** | 0.50(0.44,0.56) | <0.0001 | | 0.80(0.71,0.91) | <0.001 |

**^*^**Adjusted for adjusted for age, gender, race/ethnicity, BMI, education level, family income-poverty ratio, smoking status, alcohol status, physical activity, protein intake, UACR, eGFR, diabetes, hypertension, CVD, and hyperuricemia.
